# Randomized, Crossover and Single-Dose Bioquivalence Study of Two Oral Desogestrel Formulations (Film-Coated Tablets of 75 μg) in Healthy Female Volunteers

**DOI:** 10.3797/scipharm.1111-18

**Published:** 2012-03-01

**Authors:** María Ángeles Pena, Emilio Sanz, Silvia Francisco, Ainhara Alonso, Zurine Abajo, Izaskun Felipe, Jaume Pascual, Digna Tost, Sandra Bailac

**Affiliations:** 1 Clinical Trials Unit-LEIA Foundation, T.D.C. (nowadays, Tecnalia Research & Innovation)-Hospital Txagorritxu, José Atxotegui s/n, 01009, Vitoria, Spain; 2 Kymos Pharma Services, S.L., Baldiri i Reixac, 10-12, 08028, Barcelona, Spain

**Keywords:** Desogestrel, 3-Ketodesogestrel, Low-dose oral contraceptives, Progestogen-only pills, Bioequivalence, Adverse events

## Abstract

Despite the increase in the substitution of branded medicinal product with generic drugs, this is a controversial issue for some pharmacological groups (such as contraceptives).

The aim of the present clinical trial was to assess the bioequivalence and tolerability of two oral formulations of desogestrel.

Thirty-three healthy female volunteers participated in this randomized and two-way crossover study. During two separate experimental periods, with at least four weeks of washout period, women received a single oral dose of 75 μg of desogestrel from each of the formulations (test formulation and reference formulation). Desogestrel bioavailability was determined by the measurement of 3-ketodesogestrel plasma concentration.

Pharmacokinetic parameters were comparable and the 90% CI for the ratio of C_max_ (96.14–114.53%) and AUC_0–t_ (105.73–123.83%) values for the test and reference formulations fell within the established regulatory interval (80–125%). Both formulations were also comparable in terms of tolerability.

From the results of this study it can be concluded that test formulation (desogestrel 75 μg, Cyndea PHARMA S.L.) is bioequivalent to the reference formulation (Cerazet® 75 μg, Organon Española S.A.).

## Introduction

Oral hormonal contraception has traditionally been represented by a combination of estrogens and progestogens.

To minimize side effects, these preparations are increasingly using lower doses of sex steroids. Thus, for example, the term “mini-pill” (for the prepared oral contraceptives consisting of progestogen at low-dose) has been coined [1].

Progestogens are progesterone-like steroids capable of binding to receptors and emulate their actions. Although progesterone is the only natural progestin, its rapid metabolism in the gastrointestinal tract when administered orally determines its very low bioavailability. This prevents its use for contraception.

The mini-pill desogestrel (“progestogen-only mini-pill”) received the product license in Spain in 2002. Each pill contains 75 micrograms of desogestrel (a prodrug). Its active metabolite, etonogestrel (or 3-ketodesogestrel), is a progestogen with low androgenic activity and with high affinity for progesterone receptors [2, 3]. It belongs to low-dose oral contraceptives.

Desogestrel is a derivate of natural progesterone structurally related to levonorgestrel, which is also used as a contraceptive hormone.

At the daily dose of 75 micrograms, this synthetic progestogen has contraceptive action. It is suitable for use during breastfeeding and in women who are unable or unwilling to use estrogens [4].

From the pharmacological point of view, desogestrel contraceptive action is primarily achieved by inhibiting ovulation. Daily doses of 30, 50 and 75 pg desogestrel were shown to inhibit ovulation in all cycles. The 75 pg dose is preferred over the lower doses because it showed an almost constant inhibition of ovulation and the lowest extent of follicular development; in addition, it had a more acceptable bleeding pattern than the daily doses of 30 or 50 pg [5].

Another of its effects consists of increasing the viscosity of the cervical mucus, thus impeding sperm penetration.

The pharmacokinetic profile of desogestrel and its metabolite etonogestrel (or 3-ketodesogestrel) has been described in numerous research articles using young healthy volunteers [6–9]. In receptor binding studies, a greater affinity of the metabolite (3-ketodesogestrel) for the human progesterone receptor, compared to parent drug (desogestrel), has been shown.

Desogestrel is a prodrug, and the formation of 3-ketodesogestrel is crucial for the biological effects of the compound. Hasenack *et al.* conducted a study providing evidence that desogestrel acts via etonogestrel [7]: 10 women received 150 μg of desogestrel combined with 30 μg of ethinyl estradiol, and another 10 women received 150 μg of etonogestrel combined with 30 μg of ethinyl estradiol. Each formulation was ingested as a single dose, and serum samples were obtained. The results showed that desogestrel was undetectable in serum following treatment, while etonogestrel was present, and its AUC was essentially the same as the AUC obtained following etonogestrel treatment [7].

After oral administration of desogestrel, it is rapidly absorbed and almost quantitatively converted (about 80%) into etonogestrel or 3-ketodesogestrel [10].

After administration, desogestrel itself was detected only briefly (up to 3 hours) in very low concentrations, and similar concentrations of 3-ketodesogestrel were found in blood, whether desogestrel or 3-ketodesogestrel was administered [7].

In steady state conditions, the maximum serum levels (640 pg/mL) are reached 1.8–2 h after the tablets have been taken, and etonogestrel bioavailability is approximately 73% [10]. About 95.5–99% of etonogestrel are bound to serum proteins, mainly to the albumin, and to a lesser degree, to the sex steroid hormone-binding globulin (SHBG). Cytochrome P450 enzymes catalyze the oxidative bioactivation of desogestrel, and substantial first-pass metabolism by the gut mucosa and the liver, leading to formation of 3-ketodesogestrel, has been reported. Etonogestrel is subsequently metabolized to polar derivatives in the liver [6, 11].

Etonogestrel is eliminated with a mean half-life of approximately 30 hours, with no difference between a single dose and multiple doses. The excretion of etonogestrel and its metabolites (as free steroids and also as conjugated steroids) is carried out through urine and faeces (quotient 1.5:1). In breast-feeding women, etonogestrel is excreted by passive diffusion through the mother milk with a quotient milk/serum of 0.37/0.55 and milk drug levels of 98–144 pg/mL [12].

The aim of the present study was to compare the systemic bioavailability and the tolerability of two oral formulations of desogestrel 75 μg in healthy female volunteers.

## Results and Discussion

Thirty-three volunteers completed the study.

### Bioequivalence results

Calculated pharmacokinetic parameters of 3-ketodesogestrel for the test and reference formulations are shown in Table 1.

**Fig. 1.**
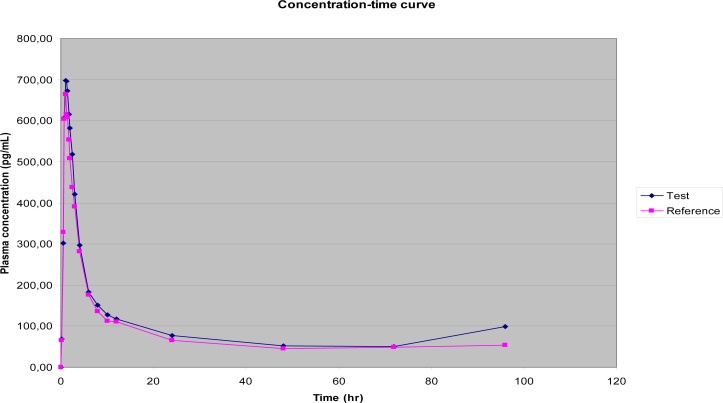
Plasma concentration-time curves of 3-ketodesogestrel.

Plasma concentration-time curves of 3-ketodesogestrel are shown in Figure 1, exhibiting the evident similarity of the plasma level profiles of both formulations.

The summary of evaluation of sequence, period and formulation effects is shown in Table 2. A significant period effect for the parameter C_max_ (p = 0.02) and significant formulation effects for AUC (p = 0.01 and p = 0.001) were found.

A summary of results of the bioequivalence analysis is shown in Table 3.

The 90% CI for the ratio of C_max_ (96.14–114.53%) and AUC_0–t_ (105.73–123.83%) values for the test and reference formulations fell within the specified bioequivalent interval (80–125%).

### Tolerability

No serious adverse events occurred during the trial. A total of 59 side-effects, in 29 women, were observed (including the analytic alterations) and these occurred. Events were recorded in 28 of the subjects with the test formulation and 31 with the reference formulation.

The most frequent related adverse events were those associated with central nervous (headache) and genitourinary systems (polymenorrhoea and dysmenorrhoea). In general, the adverse events were of mild intensity (just three adverse events were of moderate intensity).

Regarding the causal relationship, from the total number of events, 26 were not thought related to the trial drug, 23 showed a conditional relationship and 10 showed as having a causality related to the administration of some of the desogestrel study formulations (all of those were possibly related).

With respect to the analytical findings, eleven were considered as not related to the trial drug and 19 as related. Asymptomatic urinary infections predominated, representing a third of the total findings.

## Discussion

Generic medicinal products, including generic oral contraceptives, must demonstrate pharmaceutical equivalence, meaning that this new generic product (or test medicinal product) contains the same qualitative and quantitative composition in active substances and the same pharmaceutical form as the branded one (or innovator or reference medicinal product). This generic product also must be bioequivalent, which means that blood levels obtained in appropriate bioavailability clinical trials demonstrate a rate and extent of absorption not substantially different from the branded product.

If these criteria are met, the regulatory agencies do not request clinical efficacy or safety studies for the generic product before granting marketing approval, and the generic product is considered to be interchangeable with the branded product.

Moreover, brand name and generic drugs are required to conform to the same standards of Good Manufacturing Practice (GMP).

However, the bioequivalence of some generic drugs (such as oral contraceptives) continues to be a matter of controversy [13]. Women and some clinicians have questioned whether generic and branded oral contraceptives are clinically equivalent and interchangeable, effective in preventing pregnancy and equal regarding their side effects (for example, breakthrough bleeding, which is a common cause of contraceptives discontinuation) [14]. Additionally, critics point out that branded and generic oral contraceptives differ in shape, packaging, labeling, color and flavor, and claim that this could affect the treatment adherence of women and lead to unwanted pregnancies [15, 16]. Some authors, such as Goldzieher *et al*. [17], say that both oral estrogens and progestins have shown a large individual variability in the pharmacokinetics (mainly variability in hepatic first-pass) with differences in serum levels.

There are few approved generic oral contraceptives and scarce bioequivalence data published. The information regarding approved generic oral contraceptives can be seen in webs of regulatory agencies [18, 19].

Desogestrel is a prodrug and after oral administration is rapidly absorbed and converted into its active metabolite (etonogestrel or 3-ketodesogestrel). 3-Ketodesogestrel is a highly selective progestogen with low androgenic activity [3].

Due to its rapid conversion into 3-ketodesogestrel and the greater affinity of this metabolite to the human progesterone receptor, we determined the active metabolite in plasma of the female volunteers.

From the pharmacological point of view, desogestrel contraceptive action is fundamentally achieved by inhibiting ovulation.

Desogestrel pharmacokinetic profile has been described in numerous research articles. In the majority of studies with healthy female volunteers, desogestrel was administered in combination with ethinyl estradiol. Viinikka and collaborators were the first researchers who studied the pharmacokinetics of desogestrel and its pharmacological active metabolite [6].

All calculated pharmacokinetic parameter values for 3-ketodesogestrel in our study were in agreement with previously reported values in human clinical trials [20].

Traditional progestogen-only pills in comparison to combined oral contraceptives have a higher incidence of irregular menstrual bleeding. However, in a study comparing desogestrel 75 μg/day and levonorgestrel 30 μg/day there was a slightly high (not statistically significant) incidence of irregular bleeding with desogestrel (22.5% versus 18.0%) [21].

In our study, we observed eight menstrual side effects in eight women (two polymenorrhoeas, one oligomrnorrhoea and five dysmenorrhoeas). Two of the five dysmenorrhoeas were unrelated with the study medication (these women had a previous history of dysmenorrhoea and in one case the intensity was moderate). The rest of menstrual disorders observed had a possible causality with the studied medication and its intensity was mild.

## Conclusions

In summary, from the results of this study it can be concluded that test formulation (desogestrel 75 μg, Cyndea PHARMA S.L.) is bioequivalent to the reference formulation (Cerazet® 75 μg, Organon Española S.A.) with respect to its systemic bioavailability. After logarithmic-transformation, confidence intervals of the parameters C_max_ and AUC_0–t_ of 3-ketodesogestrel are within the acceptance range (80–125%). Specifically, these intervals (CI 90%) are 96.14–114.53% for C_max_ and 105.73–123.83% for AUC_0t_.

## Experimental

### Study design and ethics

The present clinical trial is a study aimed at evaluating systemic exposure to two oral formulations of a contraceptive drug.

The study design consisted of a single-centre, randomized, double-blind, two-way crossover, human pharmacology clinical trial in female volunteers.

This study was carried out in Clinical Trials Unit- LEIA Foundation, TDC (located in Txagorritxu Hospital, Vitoria-Álava, Spain).

The trial was designed according to specific national and international guidelines and in accordance with the Declaration of Helsinki and its amendments and with the ICH harmonized guideline regarding Good Clinical Practice [22–27].

The study was reviewed and approved by the local IEC (IEC at Txagorritxu hospital) and subsequently authorized by the Spanish Medicines Agency.

Written informed consent was obtained from all the participants before enrollment in the trial. The trial was registered in the EudraCT database (number: 2009-017074-20).

### Subjects

Screening was performed during nine weeks. Forty-nine healthy female volunteers were medically evaluated and thirty-four volunteers (mean age 26 years, range 18–35 years) were included (although ultimately thirty-three completed the study). All women had normal body weight and height (mean BMI: 21.43; mean weight: 58.63 kg and mean height: 165.21 cm). Prior to the study, medical history including presence of any allergy or significant disease (cardiac, hepatic, renal, pulmonary, neurological, gastrointestinal or haematological), physical examination, 12-lead electrocardiography and routine laboratory test (blood biochemistry, haematological and urinary analysis) were registered. All women were negative for hepatitis B, hepatitis C and HIV serology, drug abuse urinary test and pregnancy test. Women were not eligible to participate if clinically or analytically relevant results were identified. They were instructed to adhere to a standard protocol and were required to abstain from taking any drugs, smoking or xanthine-containing drinks consuming (including coffee and tea) for two weeks prior and during the study period. One female withdrew from the study after carrying out the first experimental period and the rest of the enrolled volunteers (33 females) completed the planned schedule.

### Study medication

Two different formulations of desogestrel 75 μg (film-coated tablets) were assayed (test formulation or formulation A: desogestrel 75 μg, Cyndea PHARMA S.L, Spain and reference formulation or formulation B: Cerazet® 75 μg, Organon Española S.A, Spain).

Following a randomized sequence balanced by blocks, women received both formulations as a single dose of 75 μg on two different experimental days, separated with a washout period of four weeks, at the least.

### Study development

Participants were admitted to the Clinical Trials Unit the evening before each experimental day, when another abuse drug test and pregnancy test were performed. The next morning, a venous catheter was inserted into a forearm vein and maintained during the session.

Women were divided into experimental groups of a minimum of 1 and a maximum of 10 individuals.

Each investigational day, study drug administration, began in the morning with 3 min intervals from one subject to another, with 200 mL of water and under the investigator’s direct surveillance. Then, they remained in relative rest in a semi-recumbent position with the head of the bed at 45-degree angle and in fasting for 4 h. Women were required to fast for 10 h before and 4 h after the drug administration.

For 3-ketodesogestrel quantification, blood samples were obtained at different times: baseline (prior to formulation administration) and 0.25, 0.5, 0.75, 1, 1.25, 1.5, 1.75, 2, 2.5, 3, 4, 6, 8, 10, 12, 24, 48, 72 and 96 h after.

Twelve hours after the administration of the formulation, volunteers were discharged from the Unit and they returned during four other occasions for 24, 48, 72 and 96 h blood extractions.

For each blood sampling, the first 1.5 mL from the catheter were ruled out, then a volume of 8 mL was taken and afterward 1.5 mL of physiological saline serum were infused to keep the venous line permeable until the next extraction.

Plasma samples were separated by centrifugation and were divided in three aliquots and later stored frozen at −20 to −80°C until its analysis in Kymos Pharma Services, S.L. (located in Barcelona, Spain).

### Tolerability assessment

Participants remained under direct surveillance by the medical staff, and safety was monitored throughout the trial.

All adverse events were immediately recorded on the individual case report form and subsequently evaluated. Vital signs (blood pressure and heart rate) were also monitored before and during the experimental session and over 96 hours after drug administration. Adverse events spontaneously experienced by the volunteers and those expressed after being asked about them during each extraction time were appropriately noted.

In addition, analytical tolerability (blood biochemistry, haematological and urinary analysis) was assessed at screening and at the end of the trial.

### Laboratory measurements

The concentrations of 3-ketodesogestrel (active metabolite of desogestrel) in human plasma (EDTA-K2) were determined according to an LC/MS/MS method validated at Kymos Pharma Services, S.L. The method validation was accomplished through determination of linearity, quantification limit, precision, accuracy, selectivity, matrix effect, dilution effect, recovery and stability.

Based on 0.7 mL of human plasma sample the internal standard (levonorgestrel) was spiked and was extracted using a solid phase extraction (Oasis® HLB-96-Well Plate 30 mg from Waters) and eluted with ethyl acetate. The extracted samples were evaporated and reconstituted in methanol:water, 40/60 (v/v). Processed samples were injected on a MDS Sciex API 4000 mass spectrometer. An XBridge C18 analytical column (5μm 4.6x50 mm from Waters) was used for chromatographic analysis. Positive ions (m/z 325.2–109.0 for 3-ketodesogestrel and 313.3–245.1 for Levonorgestrel) were monitored in the multiple reaction monitoring (MRM) mode. Linearity was assessed by using a linear regression model (1/concentration). Quantification was done by peak area ratio. This assay was validated over a nominal range of 25 to 5,000 pg/mL. Linearity over the calibration range was ≥ 0.9979. The between-run accuracy ranged from −6.17 to −0.27% with precision ranging from 6.12 to 8.85%. The within-run accuracy ranged from −8.65 to 7.00% with precision ranging from 2.42 to 11.65%. The recovery of 3-ketodesogestrel and its internal standard ranged from 70.40 to 79.76%. No matrix effect on quantitation was observed. 3-ketodesogestrel was found to be stable in human EDTA-K2 plasma after 6 h at room temperature for short term stability, after 181 days at −75±5°C for long term stability, after 82 h at room temperature for post-preparative stability and after 3 freeze and thaw cycles at −75±5°C. Dilution integrity and matrix selectivity were also demonstrated.

Study samples, for a given subject, were analyzed in a single batch. Samples were analyzed within the validated stability period (stable at least for 181 days at −75±5°C). A set of 9 non-zero calibration standards ranging from 25 to 5,000 pg/mL, blank control plasma samples and QC samples at four different concentrations (75, 250, 1,500 and 3,50 pg/mL) were prepared and analyzed in each analytical batch.

### Statistical analysis

Given the lack of literature on intra-subject variability and confidence intervals for the main pharmacokinetic parameters of the hormonal contraceptives, an approximate calculation of the sample size was decided upon.

In this sense, Timmer *et al*. reported a mean bioavailability of 0.79 for Cerazette® and 0.82 for Liseta®, with confidence intervals of 95% of 0.73–0.86 and 0.76–0.88, respectively, and with confidence intervals of 90% for AUC_0−∞_ and C_max_ of 3-ketodesogestrel between 0.89 and 1.13 and 0.91 and 1.03, respectively [20]. This three-way and cross-over bioequivalence study with one treatment arm of desogestrel only (not combined with estrogens) included data from 23 women. From these data we estimated an intra-subject coefficient of variation of 14% for AUC and 24% for C_max_, respectively.

Other published data have been obtained from bioequivalence trials by combination of ethinyl estradiol and desogestrel. For example, in FDA: Bioequivalence reviews: Center for Drugs Evaluation and Research: application number 75–256 coefficient of variation expected values (based on de data submitted in NDA for Desogen®) were 19% for C_max_ of 3-ketodesogestrel and 38% for C_max_ of ethinyl estradiol [19].

In our case, taking into account the aforementioned data and assuming that the formulations differ by 5% for a power of 90% and as a precaution against possible losses, we decided upon a total sample size of 36 subjects (with estimated maximum losses allowed of 4 subjects).

The following pharmacokinetic parameters were calculated using non-compartmental methods: the area under the curve from time 0 to the last measurable concentration (AUC_0–t_), the area under the curve from time 0 to infinity (AUC_0–∞_), maximum plasma concentration (C_max_), time to maximum plasma concentration (t_max_) and terminal elimination half-life (t_1/2_).

Plasma concentration values below the LOQ were set to zero. In accordance with the guideline on the investigation of bioequivalence, the parameters analyzed to determinate bioequivalence were C_max_ and (AUC_0–t_), and for these parameters an analysis of variance (ANOVA) was performed [22]. The ANOVA model included the evaluation of sequence, period and formulation effects at 5% level. Each ANOVA included calculation of least-squares means (LSM), the difference between formulation LSM and the standard error associated with this difference.

Firstly, a logarithmic-transformation of the parameters (with the exception of t_max_) was performed, followed by a calculation of 90% confidence interval (CI) for the ratio of the geometric means for the parameters under consideration, after the administration of each formulation. Considering the guideline on the investigation of bioequivalence, an interval of 80.00–125.00% was accepted for bioequivalence. In the case of t_max_, the 90% confidence interval for the difference of the means was calculated by the non-parametric Hauschke’s method.

Statistical analysis was performed using the WinNonlin® (Pharsight Corporation, USA) software package.

## Figures and Tables

**Tab. 1.** Pharmacokinetic parameters of 3-ketodesogestrel.

**TEST**	**t_max_ (hr)**	**C_max_ (pg/mL)**	**AUC_0–t_ (pg.hr/mL)**	**AUC_0–∞_ (pg.hr/mL)**	**t_1/2_ (hr)**

Mean	1.31	853.51	5692.05	7163.29	21.78
SD	0.60	297.35	4092.16	4912.71	9.85
Min.	0.75	295.02	1756.12	2045.53	5.36
Max.	3.00	1574.08	25305.81	31086.93	42.35
Median	1.00	727.99	4974.41	6400.09	22.11
CV (%)	46.00	34.84	71.89	68.58	45.21

**REF.**	**t_max_ (hr)**	**C_max_ (pg/mL)**	**AUC_0–t_ (pg.hr/mL)**	**AUC_0–∞_ (pg.hr/mL)**	**t_1/2_ (hr)**

Mean	1.43	818.73	5048.67	6088.36	19.21
SD	0.75	319.83	3786.31	4142.69	9.39
Min.	0.75	317.50	1186.79	1468.93	2.97
Max.	4.00	1999.73	23487.62	25892.17	40.70
Median	1.00	824.81	4359.32	5247.87	17.67
CV (%)	52.43	39.06	75.00	68.04	48.86

**Tab. 2.** Analysis of variance (α = 0.05) for the evaluation of the sequence, formulation and period effects.

	**Sequence**	**Formulation**	**Period**
**Log C_max_ (ng/mL)**	NS (p=0.25)	NS (p=0.36)	S (p=0.02)
**Log AUC_0–t_ (ng.h/mL)**	NS (p=0.38)	S (p=0.01)	NS (p=0.76)
**Log AUC_0–∞_ (ng.h/mL)**	NS (p=0.38)	S (p=0.001)	NS (p=0.70)

NS... not significant; S... significant.

**Tab. 3.** Summary of the bioequivalence analysis (n=33; 3-ketodesogestrel).

	**Arithmetic mean**		**Geometric mean**		**T/R %**	**90% CI (Classical)**

	**Reference**	**Test**	**Reference**	**Test**		
**Log C_max_ (pg/mL)**	6.64	6.69	803.44	765.65	104.94	96.14–114.53
**Log AUC_0–t_ (pg.h/mL)**	8.36	8.50	4908.23	4289.65	114.42	105.73–123.83
